# Morphology-Guided Diagnosis of Triple-Negative Breast Carcinoma: From Histopathological Evaluation to Molecular Testing

**DOI:** 10.3390/jcm15208197

**Published:** 2026-10-21

**Authors:** Valeria Zuccalà, Ida Rizzuto, Ludovica Pepe, Teresa Maria Martorana, Vincenzo Fiorentino, Pietro Tralongo, Gabriele Ricciardi, Giuliana Ciappina, Francesco Marcello Aricò, Maria Adele Marino, Massimiliano Berretta, Maurizio Martini, Guido Fadda, Giovanni Tuccari, Antonio Ieni

**Affiliations:** 1 Department of Human Pathology of Adults and Developmental Age “Gaetano Barresi”, Division of Pathology, University of Messina, Via Consolare Valeria 1, 98125 Messina, Italy; idarizzuto2@gmail.com (I.R.); ludopepe97@gmail.com (L.P.); teresa.martorana@studenti.unime.it (T.M.M.); maurizio.martini@unime.it (M.M.); guido.fadda@unime.it (G.F.); giovanni.tuccari@unime.it (G.T.); antonio.ieni@unime.it (A.I.); 2 Department of Biomedical, Dental, Morphological and Functional Imaging Sciences, University of Messina, Via Consolare Valeria 1, 98125 Messina, Italy; pietrotralongo@gmail.com (P.T.); gricciardi1998@gmail.com (G.R.); 3 Istituto Clinico Polispecialistico C.O.T. Cure Ortopediche Traumatologiche S.p.A., Via Ducezio 1, 98124 Messina, Italy; 4 Applied Biology and Experimental Medicine, Department of Chemical, Biological, Pharmaceutical and Environmental Sciences, University of Messina, 98166 Messina, Italy; giuliana.ciappina@unime.it; 5 BIOMORF Department, Oncologic Imaging Division, University Hospital Policlinico G. Martino, University of Messina, Via Consolare Valeria 1, 98125 Messina, Italy; france.arico@gmail.com (F.M.A.); mariaadele.marino@unime.it (M.A.M.); 6 Medical Oncology Unit, Department of Clinical and Experimental Medicine, University of Messina, Via Consolare Valeria 1, 98125 Messina, Italy; massimiliano.berretta@unime.it

**Keywords:** triple-negative breast carcinoma, breast pathology, morphology, immunohistochemistry, molecular pathology, differential diagnosis

## Abstract

**Background**: Triple-negative breast carcinoma (TNBC) comprises several histological entities with different molecular alterations and clinical behaviour. Recognising these entities is important for accurate classification and for choosing appropriate immunohistochemical and molecular tests. **Methods**: We conducted a narrative review of English-language literature identified through PubMed/MEDLINE, Scopus, Web of Science Core Collection, citation searching and a Scopus publication list. Journal publications available through 24 August 2026 were eligible. Current reporting and regulatory guidance was obtained from official websites. Findings were synthesised qualitatively, without statistical pooling. **Results**: The review included 93 journal publications and two official documents. Secretory carcinoma, adenoid cystic carcinoma, tall cell carcinoma with reversed polarity and metaplastic carcinoma require a different diagnostic approach from conventional high-grade invasive breast carcinoma of no special type. Architectural, cytological and stromal findings guide the differential diagnosis and the selection of immunohistochemical stains and molecular assays. Limited sampling, tumour heterogeneity and treatment-related changes can affect tissue selection and interpretation. **Conclusions**: Pathology reports should specify the histological type and clearly state any remaining diagnostic uncertainty. Morphology should guide diagnostic testing, while validated predictive biomarkers and clinical criteria determine treatment eligibility.

## 1. Introduction

Triple-negative breast carcinoma (TNBC) is conventionally defined as invasive breast carcinoma with oestrogen receptor (ER) and progesterone receptor (PR) expression in fewer than 1% of tumour-cell nuclei and without human epidermal growth factor receptor 2 (HER2) positivity according to validated immunohistochemical and in situ hybridisation (ISH) algorithms [1,2]. Although essential for clinical classification, receptor status does not establish a histological or molecular diagnosis. ER-low tumours and the HER2-low and HER2-ultralow categories illustrate the continuous nature of receptor expression and the need for defined thresholds in treatment decisions [3,4,5].

An ER-negative/PR-negative/HER2-negative profile can occur in several distinct histological entities. The current World Health Organization (WHO) classification incorporates morphology, immunophenotype, predictive biomarkers, the limitations of small specimens and recurrent molecular alterations [6]. Most TNBCs are high-grade invasive breast carcinomas of no special type (IBC-NST), but the same receptor profile also occurs in metaplastic carcinoma, secretory carcinoma, adenoid cystic carcinoma (AdCC), tall cell carcinoma with reversed polarity (TCCRP), low-grade adenosquamous carcinoma (LGASC), fibromatosis-like metaplastic carcinoma (FLMC), apocrine carcinoma, acinic cell-like carcinoma, mucoepidermoid carcinoma and rare triple-negative lobular carcinomas [6,7,8,9,10,11,12]. Several of these entities differ substantially from conventional high-grade TNBC in their clinical behaviour and molecular drivers. Acinic cell-like and mucoepidermoid carcinomas are considered in this review because, despite their variable receptor expression, they may enter the differential diagnosis of a breast carcinoma initially found to be receptor-negative [6,13,14,15,16,17]. Their classification depends on morphology and context, irrespective of triple-negative status [6,18].

Some of this biological heterogeneity can be recognised on haematoxylin and eosin (H&E) sections. Microcystic architecture with intracellular and extracellular secretory material suggests secretory carcinoma and an associated *ETV6::NTRK3* fusion [19,20]; cribriform biphasic architecture suggests AdCC and *MYB/MYBL1*-pathway activation [21,22,23]; reversed nuclear polarity strongly suggests TCCRP and recurrent IDH-pathway alterations [24,25,26,27,28]. Other morphological features are associated with broader biological phenotypes. Apocrine differentiation may indicate androgen receptor-associated biology [29,30], while high-grade syncytial growth, necrosis, pushing borders and prominent lymphocytes overlap with basal-like and BRCA-associated phenotypes [31,32]. None of these morphological associations alone establishes the corresponding molecular finding.

Triple-negative breast carcinomas show considerable genomic and transcriptional heterogeneity [32,33,34,35,36,37,38]. Common molecular features include homologous recombination deficiency, *TP53*-associated genomic instability, PI3K/AKT/PTEN and cell-cycle dysregulation, androgen receptor (AR)/luminal androgen receptor (LAR) phenotypes, and basal-like or mesenchymal transcriptional programmes [32,33,34,35,36,37,38]. Immune- and microenvironment-related programmes partly reflect differences in the composition and spatial organisation of the tumour microenvironment (TME) [39]. These features overlap and correspond only partly to conventional histological categories. Molecular findings therefore require interpretation in the context of tumour morphology.

Transcriptomic classifications have advanced the understanding of TNBC biology, but they correspond only partly to routine histological entities and are not required for everyday diagnostic classification [33,34]. A morphology-guided approach recognises the diversity of TNBC and allows ancillary tests to be selected according to the histological differential diagnosis. A recent review described the clinicopathological, molecular, prognostic and therapeutic features of rare TNBC histologies [10]. The present review examines how specific H&E findings can guide immunohistochemistry and indicate when molecular confirmation is needed.

The review addresses the principal components of the diagnostic work-up: confirmation of the triple-negative phenotype, H&E pattern recognition, focused immunohistochemistry, defining or highly characteristic molecular alterations, differential diagnosis and selected tissue-based biomarkers. The tumour microenvironment, post-neoadjuvant pathology, predictive biomarkers and digital or spatial methods are considered where they affect tissue interpretation, sampling or clinically relevant testing. The diagnostic sequence begins with H&E examination and a morphological differential diagnosis, followed by focused immunohistochemistry and, when needed, molecular confirmation to reach an integrated diagnosis.

## 2. Materials and Methods

### 2.1. Literature Search Strategy

For this narrative review, PubMed/MEDLINE, Scopus and Web of Science Core Collection were searched, with the database searches last updated on 24 August 2026. English-language journal publications were eligible from database inception through that date. Eligibility was determined by the date of first full publication, including online-first publication, irrespective of the assigned issue year. No study-design or publication-type filters were applied. Older seminal publications were included when relevant to current diagnostic practice. The same publication window and relevance criteria were applied to journal publications identified through all search methods. Guideline, reporting and regulatory information was checked against current official sources.

The three databases were searched using the same free-text terms and Boolean structure, adapted to their syntax and search fields: PubMed title/abstract-related fields [tiab], Scopus TITLE-ABS-KEY and Web of Science Topic fields (TS, including Keywords Plus). Breast or mammary carcinoma/cancer terms were combined with either triple-negative terminology or the names of special histological types and a required block of diagnostic terms. Searches for special types did not require triple-negative terminology, as relevant reports may omit receptor status from the title or abstract. The complete queries are shown below. Reference lists and forward citations of key publications were also screened for relevant reports.

**PubMed/MEDLINE**

(breast[tiab] OR mammary[tiab]) AND (carcinoma*[tiab] OR cancer*[tiab]) AND (“triple-negative”[tiab] OR “triple negative”[tiab] OR TNBC[tiab] OR “secretory carcinoma”[tiab] OR “secretory breast carcinoma”[tiab] OR “adenoid cystic”[tiab] OR “tall cell”[tiab] OR “reversed polarity”[tiab] OR “reverse polarity”[tiab] OR metaplastic[tiab] OR “fibromatosis-like”[tiab] OR “fibromatosis like”[tiab] OR adenosquamous[tiab] OR apocrine[tiab] OR “acinic cell”[tiab] OR mucoepidermoid[tiab] OR medullary[tiab]) AND (morpholog*[tiab] OR histopatholog*[tiab] OR clinicopatholog*[tiab] OR (histolog*[tiab] AND (classif*[tiab] OR subtype*[tiab] OR “special type*”[tiab] OR “rare type*”[tiab])) OR “differential diagnosis”[tiab] OR ((immunohistochem*[tiab] OR immunophenotyp*[tiab]) AND (diagnos*[tiab] OR classif*[tiab])) OR ETV6[tiab] OR NTRK3[tiab] OR MYB[tiab] OR MYBL1[tiab] OR IDH2[tiab] OR MAML2[tiab] OR NR4A3[tiab] OR TRPS1[tiab] OR GATA3[tiab] OR SOX10[tiab]) AND english[la]

**Scopus**

TITLE-ABS-KEY((breast OR mammary) AND (carcinoma* OR cancer*) AND (“triple-negative” OR “triple negative” OR TNBC OR “secretory carcinoma” OR “secretory breast carcinoma” OR “adenoid cystic” OR “tall cell” OR “reversed polarity” OR “reverse polarity” OR metaplastic OR “fibromatosis-like” OR “fibromatosis like” OR adenosquamous OR apocrine OR “acinic cell” OR mucoepidermoid OR medullary) AND (morpholog* OR histopatholog* OR clinicopatholog* OR (histolog* AND (classif* OR subtype* OR “special type*” OR “rare type*”)) OR “differential diagnosis” OR ((immunohistochem* OR immunophenotyp*) AND (diagnos* OR classif*)) OR ETV6 OR NTRK3 OR MYB OR MYBL1 OR IDH2 OR MAML2 OR NR4A3 OR TRPS1 OR GATA3 OR SOX10)) AND LANGUAGE(english)

**Web of Science Core Collection**

TS=((breast OR mammary) AND (carcinoma* OR cancer*) AND (“triple-negative” OR “triple negative” OR TNBC OR “secretory carcinoma” OR “secretory breast carcinoma” OR “adenoid cystic” OR “tall cell” OR “reversed polarity” OR “reverse polarity” OR metaplastic OR “fibromatosis-like” OR “fibromatosis like” OR adenosquamous OR apocrine OR “acinic cell” OR mucoepidermoid OR medullary) AND (morpholog* OR histopatholog* OR clinicopatholog* OR (histolog* AND (classif* OR subtype* OR “special type*” OR “rare type*”)) OR “differential diagnosis” OR ((immunohistochem* OR immunophenotyp*) AND (diagnos* OR classif*)) OR ETV6 OR NTRK3 OR MYB OR MYBL1 OR IDH2 OR MAML2 OR NR4A3 OR TRPS1 OR GATA3 OR SOX10))

English-language limits were applied using english[la] in PubMed, LANGUAGE(english) in Scopus and the Language filter in Web of Science. The database queries did not restrict publication dates, study designs or publication types. Eligibility by publication date was assessed during screening.

A publication list exported from Scopus was screened for relevance to diagnostic interpretation, ancillary testing and pathological reporting. These publications were assessed with the other search results, using the same publication window and relevance criteria. References retained for the review were checked against the statements they supported.

### 2.2. Literature Selection and Narrative Synthesis

Publications and official documents were included when directly relevant to histological classification, differential diagnosis, ancillary testing, specimen interpretation, tissue selection or pathological reporting. Receptor assessment was reviewed in relation to WHO terminology, ASCO/CAP guidance and current reporting recommendations. Genotype–phenotype associations were examined in clinicopathological series, diagnostic immunohistochemistry studies and molecular investigations. The qualitative appraisal considered study design, cohort size, pathological confirmation, assay validation and consistency across publications. Therapeutic and experimental-biology literature was included only when relevant to the selection or interpretation of tissue-based tests.

The database searches identified 1420 records: 420 from PubMed/MEDLINE, 565 from Scopus and 435 from Web of Science. After 610 duplicates were removed, 810 records underwent title/abstract screening; 642 were excluded because they fell outside the review scope or publication window. Of the 168 reports sought, eight could not be retrieved and 160 underwent full-text assessment. Eighty-six were excluded, leaving 74 journal publications. Citation searching identified 35 documents and official websites yielded two. After 18 duplicates were removed, including documents already identified in the database searches, 19 reports were sought. Three could not be retrieved; the remaining 16 were assessed and retained, comprising 14 journal publications and two official documents. The Scopus publication list contained 98 records, two of which had already been identified through the other searches. The remaining 96 records were assessed for relevance. Ninety-one did not provide sufficiently relevant evidence for the diagnostic or methodological questions addressed, and five journal publications were included.

**Figure 1:**
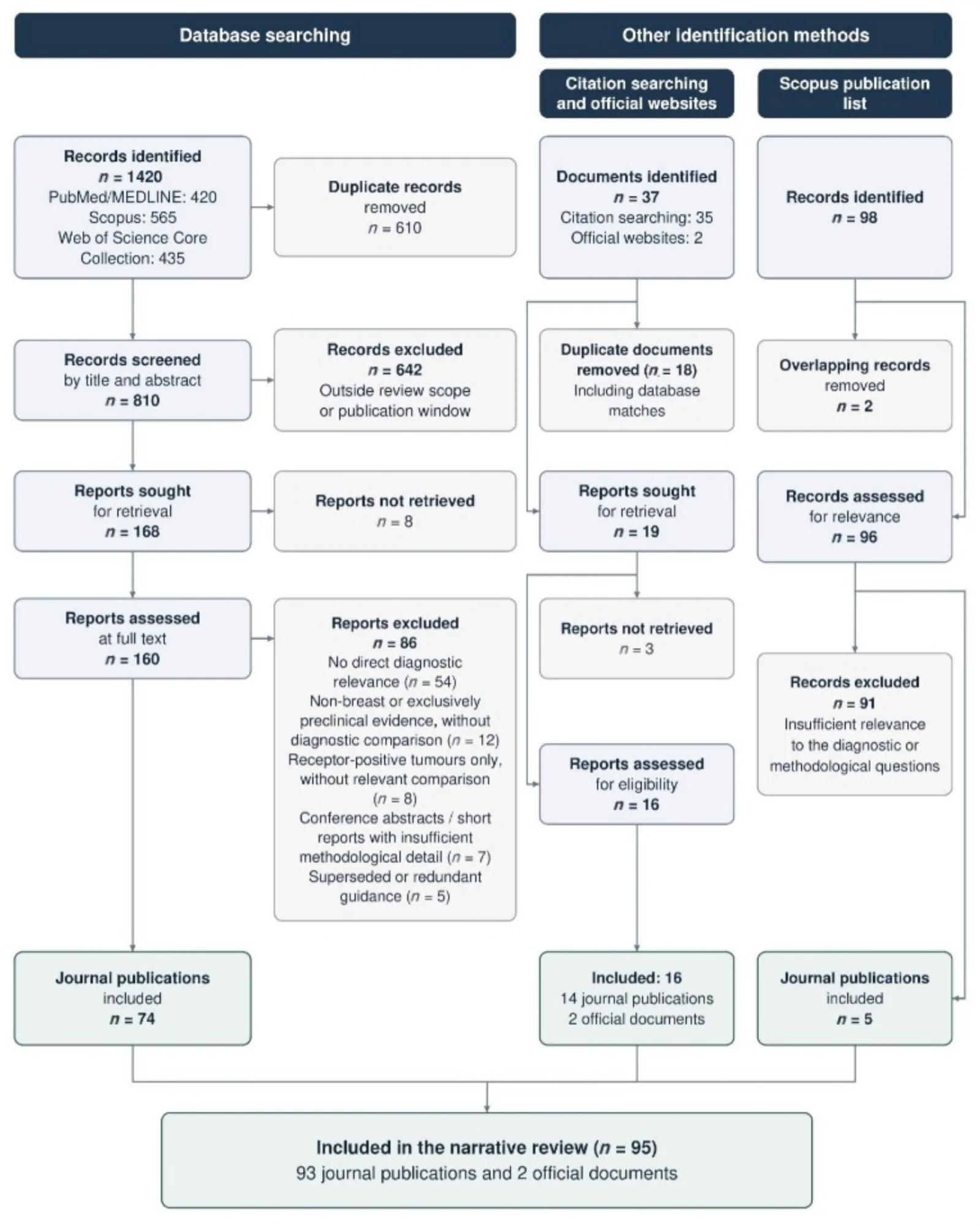
Literature identification and selection for the narrative review. The flow diagram was adapted from PRISMA 2020 [40].

One principal reason was recorded for each exclusion after full-text assessment: no direct diagnostic relevance to the review (*n* = 54); non-breast disease or exclusively preclinical evidence without a relevant diagnostic comparison (*n* = 12); evidence restricted to receptor-positive tumours without a relevant comparison (*n* = 8); retrieved conference abstracts or short reports with insufficient methodological detail (*n* = 7); or superseded or redundant guidance (*n* = 5). Reports that could not be retrieved were counted separately. The review included 93 journal publications from 2006 to 2026 and two official reporting or regulatory documents (95 in total). All are listed in the References, which also include the PRISMA 2020 methodological statement [40]. Literature identification and selection, including reasons for exclusion, are summarised in Figure 1 using a flow diagram adapted from PRISMA 2020 [40].

The review used a narrative design: no protocol was preregistered, screening was not independently duplicated, and neither a structured risk-of-bias assessment nor formal evidence grading was performed. No new statistical analysis or meta-analysis was undertaken because the publications addressed different histological entities, assays, diagnostic questions and clinical outcomes, with no common endpoint suitable for quantitative synthesis. Numerical findings are attributed to the original publications and were not pooled. Diagnostic testing establishes the tumour type or resolves a differential diagnosis; predictive testing assesses validated biomarkers relevant to treatment eligibility; exploratory testing concerns investigational profiling. The proposed diagnostic approach is based on the literature and has not been externally validated as a clinical decision rule.

### 2.3. Use of Generative Artificial Intelligence

During the preparation of this manuscript, the authors used ChatGPT, version 5.6 (OpenAI, San Francisco, CA, USA), as an auxiliary writing tool to assist with language editing, improvement of English clarity and readability, and stylistic refinement of selected parts of the text. It was also used to generate initial illustrative images for the figures, which were subsequently edited and finalised by the authors. This tool was not used to generate scientific data, perform analyses, select or interpret references, or draw scientific conclusions. The authors reviewed and edited all AI-assisted outputs, checked the final figures for scientific accuracy, and take full responsibility for the content of this publication.

## 3. Establishing the Triple-Negative Phenotype

Morphology-guided diagnosis depends on reliable ER, PR and HER2 assessment. Pre-analytical quality is particularly important in pretreatment core needle biopsy (CNB), which may provide the only untreated tissue available before neoadjuvant therapy. Cold ischaemia, fixation, crush artefact, low tumour cellularity and tissue exhaustion can cause misleadingly weak or negative staining. Internal and external controls should therefore be reviewed before a triple-negative result is accepted [1,2].

ER staining in fewer than 1% of invasive tumour-cell nuclei is interpreted as negative, whereas 1–10% staining is classified as ER-low positive [1]. ER-low carcinomas do not formally meet the TNBC definition, although some have TNBC-like biological features. An unusual ER-negative/PR-positive result warrants review of the controls and morphology, with repeat testing when appropriate. Interpretation of negative findings depends on technically adequate staining and correlation with morphology.

HER2 assessment follows the ASCO/CAP IHC–ISH algorithm: IHC 0 and 1+ are negative for overexpression, IHC 2+ is equivocal and requires ISH, and IHC 3+ is positive [2]. Reports should record the actual IHC score and, within the conventional IHC 0 category, distinguish absent membrane staining from faint, incomplete membrane staining in a small proportion of tumour cells [4,41]. The September 2026 CAP template provides separate reporting options for negative 0 with absent membrane staining and negative 0+ with membrane staining [41]. HER2-low denotes IHC 1+ or IHC 2+/ISH-negative; HER2-ultralow denotes IHC 0 with faint, incomplete membrane staining in >0% and ≤10% of tumour cells [4]. Both can occur in receptor-defined TNBC. They describe HER2 expression and do not establish distinct histological entities or independently determine treatment eligibility. Interpretation near these thresholds requires attention to controls and tissue quality, with a second review where needed [4,42].

CNB is generally reliable for determining positive or negative receptor status, but limited sampling may miss intratumoral heterogeneity, and discordant results occur more often near low-expression thresholds [43]. Distinguishing HER2 IHC 0, ultralow and low categories is more susceptible to sampling and interpretive variability [4,42,43]. Repeat testing of an analytically valid pretreatment core with concordant morphology should therefore be selective. It is most useful when the biopsy is scant or poorly fixed, ER or PR expression lies near a decision threshold, HER2 staining is heterogeneous, finer categorisation of low-level HER2 expression may affect management, or the resection contains a morphologically distinct component. Decisions to repeat testing should reflect analytical concerns, morphological discordance and clinical relevance.

Once mammary origin is considered plausible and a technically valid triple-negative receptor profile has been established, the H&E sections are reviewed in this context. The dominant morphology guides the work-up towards conventional high-grade triple-negative IBC-NST or investigation for a special histological type. Subsequent morphological, immunophenotypic or clinicoradiological discordance may prompt reassessment of mammary origin and further testing for tumour lineage. This assessment relates the receptor profile to the histological diagnosis and its biological implications.

## 4. Morphology-Guided Diagnostic Approach

**Figure 2:**
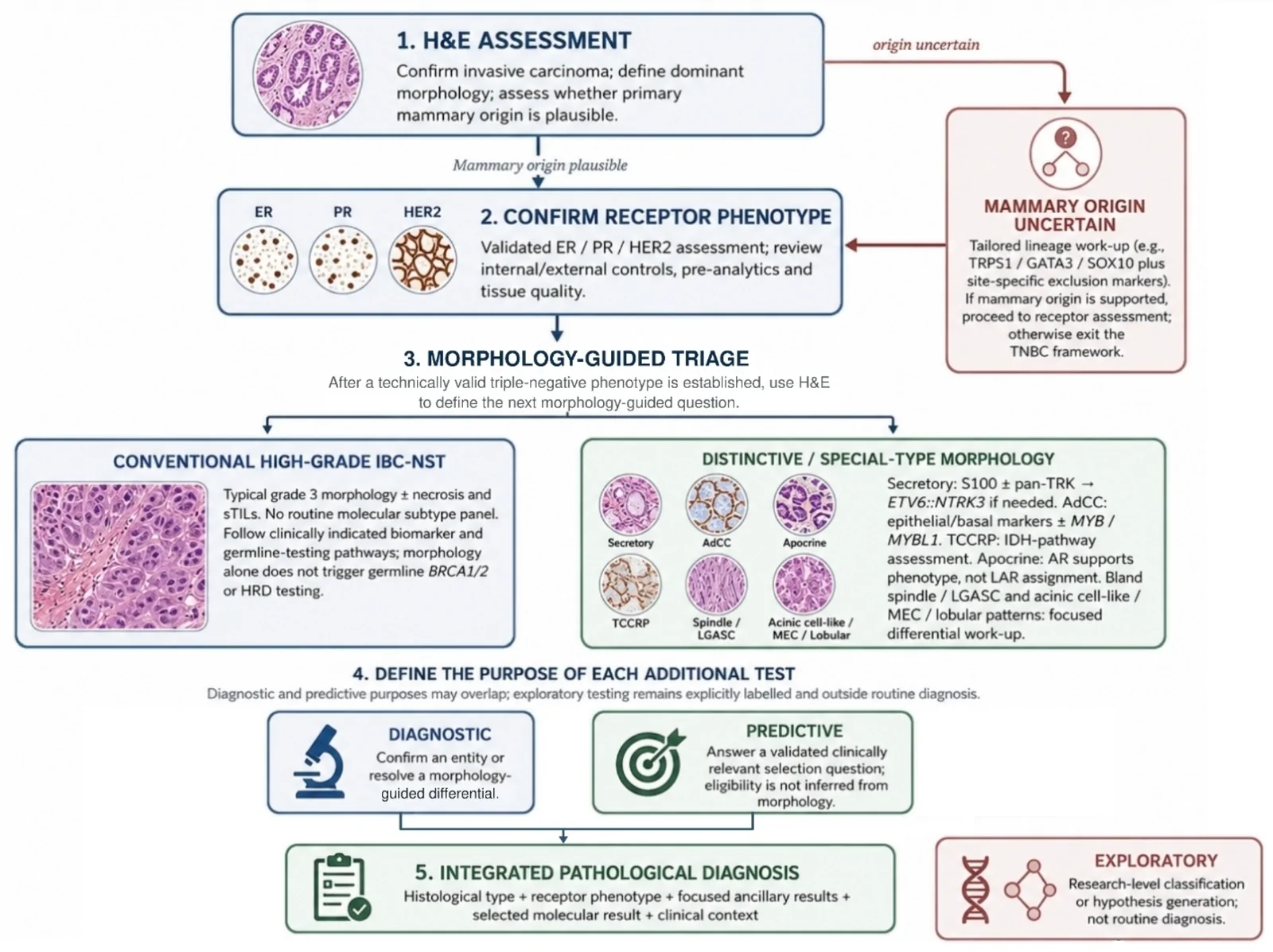
Proposed morphology-guided approach to TNBC diagnosis. H&E examination establishes invasion, dominant morphology and whether mammary origin is plausible. A tailored lineage panel is used when origin is uncertain. The TNBC approach applies once mammary origin is supported and the triple-negative phenotype is technically valid. Conventional high-grade IBC-NST generally undergoes clinically indicated biomarker assessment; distinctive morphological patterns prompt focused diagnostic testing. Ancillary findings are interpreted according to their diagnostic, predictive or exploratory purpose and incorporated into the pathology report. Morphology alone does not establish treatment eligibility. The approach is based on the literature and has not been externally validated. Histological and immunohistochemical vignettes are schematic illustrations and do not represent patient-derived micrographs. Abbreviations: AdCC, adenoid cystic carcinoma; AR, androgen receptor; ER, oestrogen receptor; GATA3, GATA-binding protein 3; H&E, haematoxylin and eosin; HER2, human epidermal growth factor receptor 2; HRD, homologous recombination deficiency; IBC-NST, invasive breast carcinoma of no special type; IDH, isocitrate dehydrogenase; LAR, luminal androgen receptor; LGASC, low-grade adenosquamous carcinoma; MEC, mucoepidermoid carcinoma; MYB, MYB proto-oncogene; MYBL1, MYB proto-oncogene like 1; pan-TRK, pan-tropomyosin receptor kinase; PR, progesterone receptor; SOX10, SRY-box transcription factor 10; sTILs, stromal tumour-infiltrating lymphocytes; TCCRP, tall cell carcinoma with reversed polarity; TNBC, triple-negative breast carcinoma; TRPS1, transcriptional repressor GATA binding 1.

H&E examination identifies the dominant architecture and cytology and establishes the differential diagnosis. Receptor testing places these findings in the triple-negative context, while ancillary tests are selected to resolve specific diagnostic questions. Special histological types are recognised primarily by morphology rather than receptor status, and occasional cases have receptor profiles outside the conventional TNBC definition. Table 1 details the morphology-guided diagnostic work-up, and Figure 2 summarises the proposed approach.

### 4.1. Conventional High-Grade Triple-Negative IBC-NST: Morphological and Molecular Features

Conventional triple-negative IBC-NST usually presents as a high-grade invasive carcinoma with limited tubule formation, marked nuclear pleomorphism, brisk mitotic activity, solid or syncytial growth, geographic necrosis and variable lymphocytic infiltration [31,32,44]. Pushing or relatively circumscribed borders may also be present [31,32]. These features overlap with the historical morphological description of basal-like carcinoma [31,32]. In a contemporary TNBC series, basal-like morphology was associated with higher grade and proliferation but was not independently associated with outcome, illustrating the limitations of prognostic assessment based on morphology alone [44].

**Figure 3:**
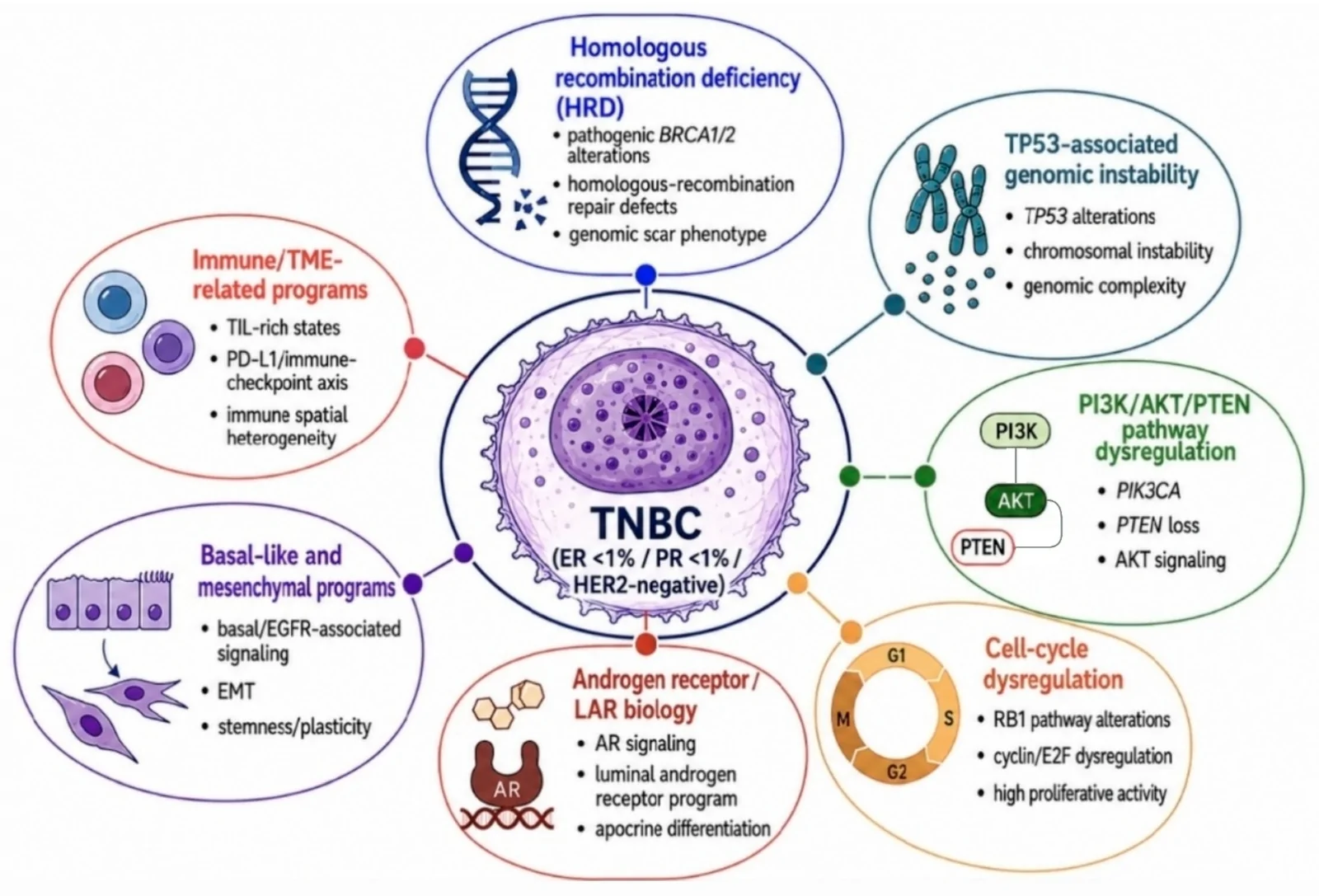
Molecular and cellular features contributing to the biological heterogeneity of TNBC. Representative molecular abnormalities, cellular states and phenotypic features are grouped according to recurrent biological processes [32,33,34,35,36,37,38,39]. These processes overlap and do not correspond to mutually exclusive histological types or molecular subtypes used in routine diagnosis. Abbreviations: AKT, protein kinase B; AR, androgen receptor; E2F, E2F transcription-factor family; EGFR, epidermal growth factor receptor; EMT, epithelial–mesenchymal transition; ER, oestrogen receptor; HER2, human epidermal growth factor receptor 2; HRD, homologous recombination deficiency; LAR, luminal androgen receptor; PD-L1, programmed death-ligand 1; PI3K, phosphoinositide 3-kinase; PR, progesterone receptor; PTEN, phosphatase and tensin homologue; TIL, tumour-infiltrating lymphocyte; TME, tumour microenvironment; TNBC, triple-negative breast carcinoma.

High-grade morphology may suggest basal-like, proliferative or DNA-repair-deficient biology, but it cannot establish these characteristics. TNBC and basal-like carcinoma overlap but are not equivalent categories [32,33,35]. *BRCA1*-associated tumours frequently show grade 3 morphology, syncytial growth, pushing margins, necrosis and prominent lymphocytes; however, these features also occur in sporadic TNBC [31,32]. Germline *BRCA1/2* status cannot be inferred from morphology [31,32,51]. The receptor-defined TNBC category encompasses several partly overlapping molecular abnormalities and cellular phenotypes, including homologous recombination deficiency, *TP53*-associated genomic instability, PI3K/AKT/PTEN and cell-cycle dysregulation, AR/LAR biology, basal-like and mesenchymal states, and variation in the immune response and tumour microenvironment. These features are summarised in Figure 3.

Histological assessment should document architecture, grade, necrosis, lymphocytic infiltration, stromal features and any spatially distinct tumour component. Cytokeratin 5/6 (CK5/6), cytokeratin 14 (CK14) and epidermal growth factor receptor (EGFR) can demonstrate basal differentiation [31,32,44], although their routine use solely to classify a conventional TNBC as “basal-like” adds little diagnostic information. Ki-67 can describe proliferative activity but is not an independent treatment-predictive classifier [32,44]. A typical grade 3 triple-negative IBC-NST without a distinctive morphological pattern generally warrants standard clinical biomarker testing rather than an experimental IHC panel intended to assign a molecular subtype.

Homologous recombination deficiency (HRD) provides a further example of the limits of morphological assessment. A “*BRCA*-like” appearance on H&E may suggest defective DNA repair, but establishing HRD requires a molecular or functional assay [32,45,46]. RAD51 foci assessment is a promising tissue-based measure of homologous recombination competence, although it is not part of routine diagnostic classification of TNBC [45,46]. In conventional high-grade triple-negative IBC-NST, morphology is therefore particularly useful for selecting tissue and recognising unusual components; it does not generally justify broad profiling solely to assign a molecular subtype.

Conventional triple-negative IBC-NST may also be morphologically heterogeneous [32,44]. Syncytial areas with abundant lymphocytes may coexist with desmoplastic, spindle-cell or apocrine-appearing regions. When a distinct component is present, the tissue selected for ancillary testing should include that component, particularly when sampling is limited to CNB or follows systemic therapy. Histological examination thus helps identify the region most appropriate for investigating a specific diagnostic or biological question.

### 4.2. IBC-NST with Medullary Pattern

Some TNBCs show syncytial growth of high-grade tumour cells, a prominent lymphoplasmacytic infiltrate and pushing borders. These tumours are classified as IBC-NST with medullary pattern rather than as a separate entity designated “medullary carcinoma” [6,9]. The pattern remains diagnostically useful because it identifies tumours with an abundant immune infiltrate. Standardised assessment of stromal tumour-infiltrating lymphocytes (sTILs) provides more useful information than attempting to assign an immunomodulatory molecular subtype from H&E [47,48]. Indications for programmed death-ligand 1 (PD-L1) testing depend on the clinical setting and cannot be determined by lymphocyte abundance alone [57,58,59].

### 4.3. Apocrine Differentiation and Androgen Receptor Expression

Apocrine morphology comprises large polygonal cells with abundant granular eosinophilic cytoplasm, sharply defined borders, enlarged nuclei and prominent nucleoli. Focal apocrine differentiation should be distinguished from pure apocrine carcinoma, a more restrictive designation. In the literature, “pure apocrine carcinoma” describes tumours with apocrine morphology in at least 90% of tumour cells and an ER-negative/PR-negative/AR-positive immunophenotype [6,29,30]. In a tumour already classified as triple-negative, ER and PR negativity are established, whereas AR immunohistochemistry provides support for the pure apocrine phenotype. GATA3 or gross cystic disease fluid protein 15 (GCDFP-15) may further support mammary or apocrine differentiation [29,30]. Triple-negative carcinomas with apocrine differentiation frequently show AR-related transcriptional features and PI3K/AKT pathway alterations. Recent clinicopathological series also indicate biological differences from conventional basal-like TNBC [30,60].

Apocrine morphology, AR positivity and the transcriptomic LAR subtype overlap but are not synonymous. AR immunohistochemistry can support an apocrine phenotype defined by morphology, but it cannot independently establish a molecular subtype. Cell cohesion should also be assessed in tumours with apocrine or histiocytoid cytology: rare triple-negative lobular carcinomas can show AR-associated biology, and subtle single-file or targetoid infiltration may otherwise be overlooked [11,12].

Triple-negative carcinomas with apocrine differentiation may have lower proliferative activity and different chemotherapy responsiveness from conventional basal-like TNBC [29,30,60]. Neither apocrine morphology nor AR expression alone, however, establishes a predictive biomarker. The diagnosis should describe the histological and immunophenotypic findings, with molecular LAR terminology reserved for tumours evaluated using an appropriate transcriptional classifier.

### 4.4. Metaplastic Carcinoma: Spindle-Cell, Squamous and Matrix-Producing Patterns

Spindle-cell or overt squamous differentiation, malignant chondroid or osseous matrix, and abrupt transitions between epithelial and mesenchymal-appearing areas suggest metaplastic carcinoma. Metaplastic carcinomas are commonly triple-negative but differ from conventional IBC-NST in morphology, differential diagnosis, molecular alterations and reported response to neoadjuvant treatment [61,62]. Recent response data support documenting histological type, although the small numbers of special-type tumours do not support treatment recommendations based on histology alone [62].

Ancillary testing should address the differential diagnosis suggested by the histology. Broad-spectrum and high-molecular-weight cytokeratins are useful because epithelial marker expression may be focal, particularly in spindle-cell areas [64,65]. p63 and p40 can support squamous or metaplastic differentiation but lack specificity; several diagnostic pitfalls are recognised for p63 in breast pathology [63]. A single negative keratin stain does not exclude metaplastic carcinoma. Conversely, isolated expression of an epithelial-type marker does not outweigh characteristic biphasic morphology in a phyllodes tumour [64,65]. When CNB findings remain indeterminate, deeper sections, additional keratin stains and assessment of the architecture in the excision specimen are often more informative than unfocused molecular testing [64,65]. Section 5.3 discusses the differential diagnosis in relation to architecture and grade. In bland spindle-cell lesions, keratin and/or p63 expression in lesional spindle cells supports FLMC over fibromatosis, scar and nodular fasciitis, which are generally negative for these markers. Staining in FLMC may be very focal and must be distinguished from entrapped benign epithelium [64,65].

### 4.5. Secretory Carcinoma and ETV6::NTRK3 Fusion

Secretory carcinoma has a well-established association between morphology and its characteristic molecular alteration. Microcystic, tubular, follicular-like or solid architecture with intracellular and extracellular eosinophilic secretory material strongly suggests the diagnosis. S100 is frequently expressed but is not specific. Strong pan-TRK staining in an appropriate distribution provides additional diagnostic support [20].

The defining molecular alteration is the *ETV6::NTRK3* fusion, which is present in the great majority of conventional secretory carcinomas [19]. Molecular confirmation is particularly useful when morphology is atypical, pan-TRK staining is equivocal, or demonstrating an NTRK fusion has diagnostic or therapeutic implications [19,20]. In a recent 29-case cohort, the metastatic tumour showed predominantly solid architecture, greater atypia, mitotic activity and necrosis, suggesting that features resembling high-grade transformation may identify rare aggressive cases [19]. Morphological suspicion can therefore be followed by S100 with or without pan-TRK staining and, when indicated, targeted fusion testing.

In small biopsies, microcystic or follicle-like architecture may be misinterpreted as nonspecific low-grade carcinoma, particularly when characteristic secretions are focal. S100 positivity alone does not establish secretory carcinoma because it occurs in several breast and non-breast tumours [20]. Pan-TRK is similarly a screening stain that requires morphological interpretation; positivity does not by itself confirm an NTRK rearrangement. Diagnostic confidence is greatest when architecture, secretory material, immunophenotype and fusion status are concordant.

### 4.6. Adenoid Cystic Carcinoma: Biphasic Morphology and MYB-Related Alterations

AdCC should be considered in a triple-negative tumour with cribriform, tubular or trabecular architecture, true lumina and pseudolumina containing basement-membrane-like material. Epithelial markers together with p63/p40 or other basal markers can support the biphasic epithelial and basal/myoepithelial phenotype [22,23,67]. MYB immunohistochemistry is particularly useful in difficult cases with solid-basaloid morphology, while molecular testing may identify *MYB::NFIB* or other *MYB/MYBL1*-related alterations [21,22,23]. These alterations can support the diagnosis, although the canonical *MYB::NFIB* fusion is not required, particularly in solid-basaloid AdCC [21,22,23].

The distinction between classic and solid-basaloid AdCC has clinical relevance. Classic cribriform AdCC generally behaves much more indolently than conventional high-grade TNBC. Solid-basaloid AdCC, however, may show higher grade, necrosis, increased mitotic activity, more aggressive behaviour and limited response to neoadjuvant chemotherapy [21,66,67]. Recent molecular series indicate that *MYB* activation may persist in solid-basaloid tumours despite the relative rarity of canonical *MYB::NFIB* fusion [21,22,23]. Histological subclassification should therefore be recorded even when molecular findings support the diagnosis.

The differential diagnosis includes cribriform ductal carcinoma in situ (DCIS), collagen spherulosis, basal-like invasive carcinoma and other salivary gland-like neoplasms [22,23,66,67]. Identifying a true biphasic cellular population and pseudolumina containing basement-membrane-like material is more informative than using an extensive panel of basal markers. MYB is particularly helpful when classic architecture is incompletely represented or the tumour is predominantly solid. Absence of the canonical *MYB::NFIB* fusion does not exclude AdCC, as alternative MYB/MYBL1-related alterations occur.

### 4.7. Tall Cell Carcinoma with Reversed Polarity and IDH-Related Alterations

TCCRP is a rare entity characterised by solid or solid-papillary architecture and tall or cuboidal cells with nuclei displaced towards the luminal pole. Nuclear grooves, intranuclear pseudoinclusions and resemblance to papillary thyroid carcinoma may be present. The differential diagnosis includes other papillary breast lesions, particularly in core biopsies. Reversed nuclear polarity, interpreted alongside the immunophenotypic and molecular findings, helps distinguish TCCRP from these lesions [24]. Clinical history and thyroid-lineage markers can help exclude metastatic thyroid carcinoma when this is a consideration.

Most TCCRPs harbour recurrent *IDH2* R172 hotspot mutations, frequently accompanied by *PIK3CA* alterations. Mutation-specific IDH2 R172 immunohistochemistry can therefore support the diagnosis [24,25,26]. The commonly used 11C8B1 antibody detects the frequent R172S/R172T substitutions but not every *IDH2* R172 variant; a small minority of morphologically typical tumours are also *IDH2* wild-type [25]. Rare tumours with otherwise typical morphology and an IDH1 hotspot mutation have also been reported [27]. A *TET2* truncating mutation with a concurrent *PIK3CA* mutation has also been reported in an *IDH2*-wild-type tumour [28]. Accurate recognition is clinically relevant because TCCRP usually has a low proliferative index and favourable behaviour despite frequently meeting the receptor-based definition of TNBC.

Reversed nuclear polarity is the principal morphological clue. Basally positioned nuclei argue against TCCRP even if an IDH-directed stain is equivocal. Conversely, negative mutation-specific *IDH2* staining does not exclude TCCRP with classic morphology, given the variant-dependent coverage of the antibody. Broader molecular testing may be appropriate in such cases [25,26,27,28]. Morphology identifies the suspected entity, while the range of associated alterations informs the choice of a confirmatory assay.

### 4.8. Bland and Other Unusual Triple-Negative Morphologies

A triple-negative carcinoma with deceptively bland or otherwise unusual morphology warrants consideration of a special histological type before extensive ancillary testing. FLMC consists of bland spindle cells arranged in long infiltrative fascicles, with low mitotic activity and minimal pleomorphism. Broad epithelial markers and p63/p40 may help distinguish it from desmoid-type fibromatosis or scar, although keratin expression can be focal. Nuclear β-catenin can support fibromatosis in the appropriate context [65,68,69]. LGASC typically comprises small infiltrative glands with variable squamous differentiation in a sclerotic stroma, often associated with changes resembling a radial scar or complex sclerosing lesion [70,71]. It may mimic a benign sclerosing lesion on CNB. Assessment of infiltrative architecture and appropriately selected epithelial and myoepithelial markers is more informative than molecular profiling in this setting [70,71].

Acinic cell-like carcinoma is another special type recognised in the current WHO classification [6]. Tumours often have abundant granular eosinophilic cytoplasm and acinar or microglandular architecture. They are not uniformly low-grade, however, and solid high-grade components with marked cytological atypia have been described [72]. The 2026 WHO classification replaced “acinic cell carcinoma” with “acinic cell-like carcinoma” because mammary tumours with this morphology generally lack the characteristic *NR4A3* rearrangement and/or NR4A3 protein overexpression of salivary-gland acinic cell carcinoma. Their molecular profile instead resembles conventional TNBC and carcinomas associated with microglandular adenosis [6]. Recent population-level data also argue against assuming a uniformly triple-negative or indolent phenotype. In a 149-case National Cancer Database analysis, ER expression was reported in 57.1%, PR expression in 50.7%, 44.6% of tumours were poorly differentiated or anaplastic, and 27.0% were node-positive [13]. Registry classification without central histological review cannot redefine the entity, but these findings emphasise the need to assess morphology independently of receptor status and presumed low grade. Testing for *NR4A3* rearrangement and/or NR4A3 protein expression may be useful when a salivary primary is a plausible differential diagnosis. Mammary acinic cell-like carcinoma remains a diagnosis based on morphology and clinical context, without a requirement for a salivary-type molecular alteration.

Mucoepidermoid carcinoma (MEC) of the breast comprises mucinous, intermediate and squamoid/epidermoid cells arranged in variable solid and cystic patterns [14,15,16,17,18]. Low-grade tumours predominate in published series, but receptor expression varies and a triple-negative phenotype is not required for diagnosis. Small molecular studies have reported discordant findings. Pareja et al. identified *CRTC1::MAML2* in the single breast MEC they analysed [14]. In a four-case 2024 series, *MAML2* rearrangement was detected in three tumours, with *CRTC1::MAML2* confirmed by RNA sequencing in two [15]. A separate 13-case cohort found no MAML2 or EWSR1 rearrangements in the nine cases described in its FISH results [16], whereas both additional breast MECs reported in 2026 showed *MAML2* rearrangement [17]. A *MAML2* rearrangement or *CRTC1::MAML2* fusion therefore provides strong diagnostic support when the morphology is compatible. A negative result does not exclude breast MEC, and the frequency of these rearrangements and fusions in mammary MEC remains uncertain.

Departure from the usual high-grade TNBC morphology should prompt consideration of a special histological type. Bland cytology, distinctive architecture, granular eosinophilic cytoplasm, mixed mucinous-squamoid cells or a characteristic relationship with the stroma warrant focused investigation before classification as triple-negative IBC-NST. A limited panel selected for the specific differential diagnosis may provide more information than a broad “TNBC panel” while conserving tissue.

### 4.9. Discohesive or Histiocytoid Morphology: Lobular Carcinoma

Rare triple-negative invasive lobular carcinomas may show pleomorphic, apocrine or histiocytoid cytology. Single-file infiltration, targetoid growth, discohesive cells and intracytoplasmic lumina should prompt assessment of E-cadherin and the catenin complex. Aberrant staining patterns can occur, and immunohistochemical findings require interpretation in relation to the morphology [6,11,12]. A triple-negative phenotype does not remove the need to assess cell cohesion.

## 5. Differential Diagnosis and Ancillary Testing

### 5.1. Poorly Differentiated Carcinoma: Establishing Breast Origin

Typical primary breast tumours with concordant imaging usually do not require additional confirmation of mammary origin. Such confirmation becomes important in metastatic disease, axillary or distant-site biopsies, poorly differentiated carcinomas with an unusual phenotype, and tumours with atypical morphology or clinicoradiological findings. The absence of an in situ component may increase uncertainty but does not, by itself, indicate metastasis. Mammaglobin and GCDFP-15 have limited sensitivity in TNBC. GATA3 remains useful, although its expression may be reduced in high-grade TNBC. TRPS1 and SOX10 improve sensitivity in selected settings, although neither is entirely specific [49,50]. When lineage is uncertain, the immunophenotypic profile must be considered alongside the clinical and imaging findings [49,50]. Imaging of suspicious regional nodes provides complementary anatomical and N-staging information [75].

An initial panel for a tumour of uncertain breast origin may include TRPS1 + GATA3 ± SOX10, with additional markers selected for the competing diagnosis [49,50]. TRPS1 can be expressed in non-mammary malignancies, while SOX10 also marks melanoma and several salivary and neural crest tumours [50,73,74]. A positive breast-lineage marker therefore needs to be interpreted as part of the overall immunophenotype, with attention to morphology and the clinical setting.

A small panel of sensitive breast-lineage markers can be combined with markers specific for the suspected alternative site [49,50,73]. For example, a high-grade carcinoma in a patient with an adnexal mass warrants comparison of breast and Müllerian markers. In a patient with a lung mass, breast-lineage markers can be assessed alongside thyroid transcription factor-1 (TTF-1) and Napsin A if adenocarcinoma is suspected. This allows testing to address the differential suggested by the morphology and clinical findings.

### 5.2. Müllerian, Pulmonary, and Melanocytic Mimics

Distinguishing TNBC from high-grade serous Müllerian carcinoma can be difficult in patients with *BRCA*-associated cancer susceptibility or disseminated disease. PAX8 and WT1 support Müllerian differentiation, whereas TRPS1/GATA3/SOX10 support breast origin, but none should be interpreted without considering recognised pitfalls [50,73]. In a comparative series, TRPS1 was highly sensitive for TNBC but was also expressed in a substantial subset of high-grade serous carcinomas [73]. PAX8 positivity in TNBC was strongly clone-dependent: staining occurred with MRQ50 but not with BC12 in that study [73]. When this differential is relevant, the PAX8 clone should be documented and its result interpreted with the rest of the panel [73]. p53 is usually unhelpful because abnormal staining patterns are common in both tumour types [73]. Morphology, antibody clone, imaging and clinical history remain essential to interpretation.

Poorly differentiated lung carcinoma may also enter the differential. TTF-1 and Napsin A support pulmonary adenocarcinoma, whereas p40/p63 indicate squamous differentiation without establishing the site of origin, since metaplastic squamous breast carcinoma may express the same markers [50,63]. Melanoma is another potential mimic. SOX10 and S100 may be positive in TNBC [49,50,74], and rare melanocyte colonisation can complicate the interpretation of superficial or pigmented breast carcinomas [74,76]. In an amelanotic poorly differentiated tumour, cytokeratins, breast-lineage markers and melanocytic markers should be assessed together [49,50]. Dedifferentiated melanoma may lose conventional melanocytic marker expression, making the clinical history particularly important when the panel is discordant [77].

### 5.3. Spindle-Cell Lesions

Distinguishing spindle-cell metaplastic carcinoma from malignant phyllodes tumour, primary sarcoma, fibromatosis and reactive spindle-cell lesions is particularly difficult on breast CNB. The initial assessment should establish whether the lesion is biphasic or monophasic and low or high grade [65]. Leaf-like architecture, periductal stromal condensation, benign epithelial elements and stromal overgrowth support phyllodes tumour. A conventional carcinoma component, squamous differentiation, DCIS or convincing epithelial differentiation within malignant spindle cells supports metaplastic carcinoma.

Limited sampling may prevent a definitive diagnosis. A high-grade spindle-cell lesion can be reported as a malignant spindle-cell neoplasm with a favoured differential when the necessary architecture is not represented. A bland keratin-negative lesion should not be classified as fibromatosis until FLMC has been considered and the clinicoradiological findings are concordant. Immunohistochemistry can narrow the differential but cannot compensate for inadequate sampling [64,65].

An isolated immunohistochemical result should not outweigh convincing architecture. Keratin expression in metaplastic carcinoma may be extremely focal [64,65], and focal cytokeratin or p63 expression can also occur in malignant phyllodes tumours [64,65]. Bland FLMC may be difficult to distinguish from fibromatosis on a limited core. If morphology and IHC remain discordant, a descriptive diagnosis with classification deferred to excision may be more appropriate than assignment to a specific entity [64,65].

### 5.4. Salivary Gland-like Tumours and Site of Origin

Secretory carcinoma and AdCC share recurrent pathway alterations with morphologically similar tumours arising at other anatomical sites. In tumours with compatible morphology, *ETV6::NTRK3* supports secretory carcinoma and *MYB/MYBL1*-pathway alterations support AdCC, but neither finding alone establishes mammary origin [18]. Conversely, the morphological resemblance of mammary acinic cell-like carcinoma to salivary acinic cell carcinoma does not imply shared NR4A3-driven biology [6]. Ancillary markers also need to be interpreted in relation to the site: variable DOG1 immunoreactivity in salivary acinic cell carcinoma does not establish its diagnostic performance in mammary acinic cell-like carcinoma [78]. The molecular findings in breast MEC are less consistent. *CRTC1::MAML2* or *MAML2* rearrangement has been demonstrated in small breast series and recent cases, including 1/1, 3/4 and 2/2 tested tumours, respectively [14,15,17]. A separate 13-case cohort reported no *MAML2* or *EWSR1* rearrangements in the nine cases described in its FISH results [16]. Establishing primary breast origin therefore still requires concordant anatomical and clinicopathological findings [18].

### 5.5. Core Needle Biopsy and Tissue Stewardship

Limited architecture and tissue availability make careful test selection particularly important on CNB [64,65]. H&E examination should establish the plausible differential before a focused ancillary panel is chosen. Repeated broad panels may deplete material needed for ER/PR/HER2 reassessment, PD-L1, ISH, fusion testing or sequencing. Selective deeper levels can reveal an epithelial component, secretory material, cribriform architecture or a relationship with benign ducts, sometimes resolving the diagnosis without further biomarker testing.

Assessment of tissue adequacy should accompany diagnostic planning. If a cytology cell block is used for ancillary testing, its cellularity must also be reviewed [79]. When molecular confirmation is likely to be required, testing should use a representative block. In a heterogeneous tumour, the selected region should contain the morphological component that prompted the test [64,65].

## 6. Tumour Microenvironment and Morphological Interpretation

sTIL assessment is the best-established standardised morphological measure of the TNBC microenvironment [39,47,48]. The score represents the percentage of stromal area within the invasive tumour occupied by mononuclear inflammatory cells. Necrosis, DCIS, biopsy-related inflammation and tissue outside the invasive tumour are excluded; reporting a continuous percentage is preferred to qualitative categories [47,48].

Recent studies support the prognostic relevance of sTILs and their association with pathological response, including in contemporary chemo-immunotherapy cohorts [52,53]. These findings support careful sTIL assessment but do not establish a stand-alone threshold for withholding systemic treatment or selecting immunotherapy. The report should give the measured stromal percentage separately from any interpretation of its clinical significance.

Spatial organisation may provide information beyond the sTIL percentage. Tertiary lymphoid structures (TLS), immune-cell infiltration of tumour nests and patterns of immune exclusion can help identify representative regions for research assays [54,55,56,80]. In routine reports, these findings should remain descriptive and should not be used as definitive predictors of treatment response or as a reason for additional immune-marker panels. Tissue selection should account for heterogeneity and avoid necrotic or otherwise unrepresentative regions.

## 7. Post-Neoadjuvant Pathology

Examination of post-neoadjuvant specimens establishes the extent of treatment response and residual disease. Pathological complete response (pCR) is strongly associated with outcome, while Residual Cancer Burden (RCB) quantifies residual disease using tumour-bed dimensions, residual cellularity, in situ proportion, nodal burden and the largest nodal metastasis [81,82]. RCB retains prognostic value in contemporary chemo-immunotherapy settings [83]. Reports should document residual tumour burden and nodal involvement and compare the residual morphology with the pretreatment diagnosis. In a retrospective chemo-immunotherapy cohort, metaplastic carcinoma had lower pCR rates than IBC-NST. The small triple-negative lobular subgroup had an observed pCR rate of 56.2%, compared with 65.4% in IBC-NST, and an exploratory association with less favourable event-free survival [62].

Comparison with the pretreatment specimen can clarify the residual morphology. Treatment may preserve the original pattern or reveal a previously minor spindle, apocrine, discohesive or other distinctive component, prompting selective biomarker reassessment or sampling for molecular testing. Residual sTILs may provide prognostic information beyond residual burden [84], and HER2 IHC categorisation can differ between pretreatment and residual tumour [85]. Selective reassessment may be appropriate when the morphology changes, the pretreatment sample was limited or a new result could affect a clinically relevant treatment option.

## 8. Biomarkers and Their Implications for Pathological Testing

Diagnostic testing establishes the tumour type or resolves a differential diagnosis, whereas predictive testing determines whether a validated biomarker criterion is met in a specified clinical setting. Morphology can help select diagnostic tests. Treatment eligibility requires the appropriate biomarker result together with the relevant clinical criteria.

**PD-L1.** The indication for testing depends on the clinical setting, not on lymphocyte abundance. For advanced TNBC indications requiring PD-L1 22C3 pharmDx selection, the relevant threshold is a Combined Positive Score (CPS) ≥10 [57,86]. The report should state the assay, platform and scoring method and give the numerical CPS. Assessment requires sufficient viable tumour, appropriate controls and correct attribution of staining to tumour cells, lymphocytes and macrophages. Different assays and scoring systems should not be assumed to be interchangeable [58,87]. PD-L1 positivity is not required in the established high-risk early-TNBC perioperative setting [59]. A high sTIL percentage therefore does not replace PD-L1 testing or establish immunotherapy eligibility.

***BRCA1/2* and HRD.** Germline *BRCA1/2* pathogenic or likely pathogenic variants have established predictive relevance in specified clinical settings [51,88]. Referral should follow clinical criteria rather than depend on “*BRCA*-like” morphology, and variants of uncertain significance should not guide management [51]. Germline status requires dedicated genetic testing and cannot be inferred from the tumour phenotype. Genomic HRD scores and functional RAD51 assays assess related but distinct aspects of DNA-repair biology and do not substitute for germline *BRCA1/2* status. RAD51 assays remain promising, but evidence is insufficient to establish routine HRD-based treatment selection in TNBC [45,46,89].

**HER2 expression.** The IHC score, membrane-staining pattern and ISH result should be recorded even when the tumour is HER2-negative under the conventional algorithm [2,4,41]. HER2-low expression may be clinically relevant in advanced hormone receptor-negative disease [90]. The pivotal evidence for treatment selection in HER2-ultralow disease concerns hormone receptor-positive metastatic breast cancer and should not be extrapolated automatically to TNBC [91]. The measured result should be reported clearly, with its therapeutic interpretation determined by the disease setting and validated eligibility criteria.

**NTRK and TROP2.** Secretory morphology supports targeted testing for *ETV6::NTRK3*, which may have both diagnostic and predictive significance [19,20]. Pan-TRK IHC can be used for screening but does not, by itself, demonstrate a therapeutically relevant fusion. The current TNBC indication for TROP2-directed therapy does not require tumour TROP2-expression testing, and reflex TROP2 IHC has no established role in the diagnostic panel [86,89].

## 9. Interpreting the Pathology Report in Clinical Practice

The pathology report should make clear whether a triple-negative tumour is conventional IBC-NST or a special histological type, explain any diagnostic uncertainty and distinguish findings that establish tumour type from treatment-predictive biomarkers [6,9,10]. Table 2 summarises the histology-specific information most useful to medical oncologists.

Regardless of histological type, the report should include ER and PR results, the HER2 IHC score and ISH result when applicable, specimen site and treatment context, histological grade and stage-relevant findings [1,2,41]. After neoadjuvant therapy, residual burden and nodal findings should be reported, with comparison to pretreatment morphology where informative [81,82,83,84,85]. An uncertain special type, discrepant receptor result or unusual residual component should prompt discussion with the pathologist before further testing depletes the available tissue. Histological type informs risk assessment and the diagnostic work-up, while treatment planning requires clinical stage and validated biomarkers.

## 10. Digital and Spatial Pathology

Digital and spatial approaches may help quantify sTILs, tumour–stroma relationships, immune-cell localisation and intratumoral heterogeneity, and help select regions for confirmatory testing [92,93,94,95]. Their potential value in diagnostic practice depends on reliable measurement, sound interpretation and representative tissue selection.

Spatial studies in TNBC, including X-SPATIO, link H&E-defined regions with spatial biomarker expression. Spatial transcriptomic investigations also demonstrate different molecular and immune programmes across morphologically distinct regions [93,94,95]. These observations may guide sampling for research assays, but do not establish a routine alternative to tissue-based biomarker testing.

Computational prediction of biomarkers or treatment response from H&E remains investigational. Clinical implementation requires independent validation across laboratories and representative patient populations, with attention to technical variation and dataset bias [92,96]. Model predictions cannot replace validated receptor, PD-L1 or molecular assays.

## 11. Limitations and Future Directions

The English-language restriction and purposive selection of literature may have introduced selection bias. The search queries, selection counts and main reasons for exclusion are reported, but no protocol was preregistered, screening was not independently duplicated, and neither a structured risk-of-bias assessment nor formal evidence grading was performed. The proposed diagnostic approach is based on a qualitative synthesis and has not been validated as a diagnostic algorithm. Evidence for several rare histological types comes from small retrospective series or molecular case studies. Assay performance may also vary with antibody clone, platform, specimen type and pre-analytical conditions. Questions remain about the reproducibility and clinical utility of HER2-low/ultralow reporting and H&E-based biomarker prediction. Future studies should assess interobserver agreement for morphological findings that prompt testing, the additional diagnostic yield of focused ancillary panels, and their effects on tissue consumption, turnaround time and cost. Prospective multicentre evaluation and independent validation of computational models are needed before these approaches can support additional clinical decisions.

## 12. Conclusions

A TNBC pathology report should identify the histological type, document reliable receptor results and explain any unresolved diagnostic uncertainty. Distinctive architecture and cytology should guide the choice of ancillary tests. Conventional high-grade IBC-NST does not require routine molecular subtype assignment.

Findings that confirm tumour type should be distinguished from biomarkers used to select treatment. Detection of an NTRK fusion in secretory carcinoma can support the diagnosis and identify a potential therapeutic target. Treatment selection also depends on the clinical setting and the applicable eligibility criteria.

Diagnostic and predictive tests should be planned together to conserve tissue, particularly in small biopsies. Any specimen limitations that affect interpretation should be clearly stated in the report.

## Figures and Tables

**Table 1:** Morphology-guided differential diagnosis and ancillary testing in TNBC.

Key H&E Features	Diagnostic Consideration	Selected IHC and Ancillary Tests	Molecular Findings and Diagnostic Relevance	Diagnostic Pitfalls and Practical Considerations
High-grade solid/syncytial IBC-NST; pleomorphism; mitoses; necrosis ± sTILs	Conventional triple-negative IBC-NST; basal-like/HRD-enriched biology possible [31,32,44,45,46]	Routine IHC/molecular subtype panels are generally not required for classification; use lineage markers when mammary origin is uncertain or questioned; quantify sTILs when relevant [32,44,47,48,49,50]	Predictive/genetic assessment: germline *BRCA1/2* testing follows clinical/genetic criteria and does not define the histological type [51]. Investigational: HRD/RAD51 assays do not define a histological entity [45,46].	Basal-like or *BRCA*-like morphology is probabilistic, not a molecular diagnosis [31,32,45,46].
Lymphocyte-rich/medullary pattern	IBC-NST with medullary pattern [6]; immune-rich phenotype [47,48,52,53,54,55,56]	Standardised sTIL assessment [47,48]; recent evidence on prognosis and pCR [52,53]; PD-L1 22C3 CPS only when clinically indicated, independently of morphology [57,58,59]	No established entity-defining molecular alteration or routine molecular confirmation.	High sTILs alone do not establish immunotherapy eligibility; lymphocyte-rich morphology alone is not an indication for PD-L1 testing [57,58,59].
Extensive apocrine morphology (≥90% in “pure” definitions): eosinophilic granular cells, sharp borders, prominent nucleoli	Apocrine differentiation/”pure” apocrine phenotype; AR-related phenotype [6,29,30,60]	AR ± GATA3/GCDFP-15; AR supports the “pure” phenotype [29,30]	No entity-defining molecular test. PI3K/AKT alterations are associated with the apocrine phenotype but are non-specific and do not establish apocrine carcinoma or LAR [30,60].	Focal apocrine differentiation does not establish the “pure” phenotype; apocrine morphology, AR positivity and the molecular LAR subtype are not synonymous [29,30,33,34].
Spindle, squamous, chondroid/osseous or matrix-producing areas	Metaplastic carcinoma [61,62,63,64,65]	Broad keratin panel ± CK5/6, p63/p40 [63,64,65]	No established entity-defining molecular test. Broad profiling does not establish the histological type and is guided by a separate clinical indication [61,64,65].	Keratin may be focal; p63 is not specific. Exclude phyllodes tumour, sarcoma, fibromatosis and melanoma [63,64,65].
Microcystic/tubular/follicular-like pattern with intracellular/extracellular secretion	Secretory carcinoma [19,20]	S100 ± pan-TRK [20]	Entity-defining when morphology is compatible: *ETV6::NTRK3* fusion [19,20]. Fusion confirmation may also have predictive relevance.	Characteristic morphology-genotype association; an NTRK fusion may also be actionable [19,20].
Cribriform/tubular biphasic tumour; true lumina + pseudolumina with basement-membrane material	Adenoid cystic carcinoma [21,22,23,66,67]	Epithelial/basal markers ± MYB [22,23,67]	Strongly supportive: MYB/MYBL1-pathway alteration. The canonical *MYB::NFIB* fusion is not required, particularly in solid-basaloid disease [21,22,23].	Specify classic or solid-basaloid morphology; *MYB::NFIB* is less consistently detected in solid-basaloid disease [21,22,23,66,67].
Solid/solid-papillary tall cells with apically displaced nuclei (reversed polarity)	Tall cell carcinoma with reversed polarity [24,25,26,27,28]	*IDH2* R172 mutation-specific IHC where validated; thyroid markers if needed [24,25]	Strongly supportive: *IDH2* R172 mutation, although not obligatory. *PIK3CA* is non-specific; rare IDH1/TET2 findings have limited evidence and are not routine diagnostic criteria [24,25,26,27,28].	Consider papillary breast lesions and thyroid metastasis; negative mutation-specific IHC does not exclude the diagnosis when morphology is typical [24,25,26,27,28].
Bland infiltrative spindle fascicles; low mitotic activity	Fibromatosis-like metaplastic carcinoma [65,68,69]	Broad keratins ± p63/p40; β-catenin when fibromatosis is in the differential diagnosis [65,68,69]	No established entity-defining molecular test [65,68,69].	Distinguish from desmoid-type fibromatosis and scar; keratin can be very focal [65,68,69].
Small infiltrative glands + squamous differentiation in sclerotic/radial-scar-like stroma	Low-grade adenosquamous carcinoma [70,71]	Targeted epithelial/myoepithelial panel as needed [70,71]	No established entity-defining molecular test [70,71].	Can mimic benign sclerosing lesions; do not extrapolate the behaviour of conventional high-grade TNBC to this entity [70,71].
Granular eosinophilic cells; acinar/microglandular architecture ± solid/high-grade component	Acinic cell-like carcinoma [6,13,72]	Breast-lineage markers as needed; *NR4A3* protein IHC ± *NR4A3* rearrangement testing when salivary acinic cell carcinoma is in the differential diagnosis [6,13,72]	No established entity-defining fusion in the breast. *NR4A3* testing is used in the differential diagnosis of salivary acinic cell carcinoma, not to confirm a breast-specific entity [6,72].	Do not assume a uniformly low-grade or triple-negative phenotype; histological/receptor heterogeneity and the microglandular adenosis-associated spectrum are relevant [6,13,72].
Mixture of mucinous, intermediate and squamoid/epidermoid cells; solid/cystic pattern	Mucoepidermoid carcinoma [14,15,16,17]	Targeted keratins/p63/p40 and mucin stains according to morphology [14,15,16,17,18]	Strongly supportive when present: *MAML2* rearrangement/CRTC1::MAML2 fusion; absence does not exclude breast MEC [14,15,16,17].	A positive *MAML2* rearrangement/fusion strongly supports the diagnosis when morphology is compatible; a negative result does not exclude breast MEC [14,15,16,17].
Discohesive, single-file, targetoid, histiocytoid/apocrine cells	Triple-negative invasive lobular carcinoma [11,12]	E-cadherin and catenin-complex assessment [6,11,12]	No entity-defining molecular test is required; classification is based on morphology and cell-cohesion assessment [11,12].	Receptor phenotype does not replace assessment of cell cohesion [11,12].
Poorly differentiated carcinoma; morphology or clinicoradiological presentation atypical for a breast primary	Metastatic tumour versus primary breast carcinoma [18,49,50,73,74]	Tailored lineage panel; e.g., TRPS1 + GATA3 ± SOX10 plus site-specific exclusion markers [18,49,50,73,74]	Site-specific molecular testing depends on the clinical and pathological findings; no universal molecular test establishes breast origin [18,49,50,73,74].	Absence of an in situ component does not establish metastasis. TRPS1 and PAX8 have important site- and clone-specific pitfalls [18,49,50,73,74].

Molecular findings are classified according to their diagnostic role: “entity-defining” denotes a characteristic driver in a tumour with compatible morphology; “strongly supportive” denotes a finding that increases diagnostic confidence but is not required for diagnosis; “investigational” denotes testing without established routine diagnostic utility. These terms apply within the appropriate morphological and clinical context. They do not imply that testing is mandatory in every case or that a molecular finding independently establishes treatment eligibility. Abbreviations: AKT, protein kinase B; AR, androgen receptor; CK5/6, cytokeratin 5/6; CPS, Combined Positive Score; GCDFP-15, gross cystic disease fluid protein 15; H&E, haematoxylin and eosin; HRD, homologous recombination deficiency; IBC-NST, invasive breast carcinoma of no special type; IHC, immunohistochemistry; LAR, luminal androgen receptor; MEC, mucoepidermoid carcinoma; pan-TRK, pan-tropomyosin receptor kinase; pCR, pathological complete response; PD-L1, programmed death-ligand 1; PI3K, phosphoinositide 3-kinase; sTILs, stromal tumour-infiltrating lymphocytes; TNBC, triple-negative breast carcinoma.

**Table 2:** Histology-specific information in TNBC pathology reports for medical oncologists.

Histological Type or Pattern	Relevant Report Findings	Interpretation and Practical Implication
Conventional high-grade IBC-NST	Grade; receptor results; clinically indicated predictive biomarkers [1,2,32,51,57]	Triple-negative status does not establish a basal-like subtype or *BRCA*/HRD status. Apply validated clinical and biomarker criteria.
IBC-NST with medullary pattern	Medullary/lymphocyte-rich pattern; sTIL percentage if assessed [6,47,48,57,59]	Immune-rich morphology is relevant to interpretation but does not establish PD-L1 positivity or immunotherapy eligibility.
Apocrine differentiation	Extent of apocrine morphology; AR result where performed [29,30,60]	Apocrine morphology, AR expression and molecular LAR are not interchangeable. AR positivity alone is not a validated routine biomarker for treatment selection.
Metaplastic carcinoma	Histological components and diagnostic confidence, especially on core biopsy [61,62,63,64,65]	Treatment response may differ from IBC-NST. Histology alone does not justify omitting clinically indicated treatment.
Secretory carcinoma	Fusion status if tested; diagnostic confirmation; high-grade features [19,20]	A confirmed NTRK fusion can have diagnostic and treatment implications in the appropriate setting. Pan-TRK alone does not confirm a fusion.
Adenoid cystic carcinoma	Classic versus solid-basaloid pattern; grade and adverse features [21,22,23,66,67]	Solid-basaloid tumours should not be assumed to share the generally indolent behaviour of classic disease. Absence of *MYB::NFIB* does not exclude the diagnosis.
Tall cell carcinoma with reversed polarity	Reversed polarity; confirmatory findings; *IDH2* result if available [24,25,26,27,28]	Confirm special-type classification before making clinical inferences from triple-negative status. Negative mutation-specific IHC does not exclude the diagnosis when morphology is typical.
Fibromatosis-like metaplastic carcinoma	Epithelial differentiation; infiltrative extent and margins [65,68,69]	A bland spindle tumour may still be carcinoma. Distinguish it from fibromatosis and assess local extent.
Low-grade adenosquamous carcinoma	Invasive glandular/squamous components; extent and margins [70,71]	Receptor negativity does not imply the behaviour of conventional high-grade TNBC. Resolve diagnostic uncertainty on limited biopsies.
Acinic cell-like carcinoma	Grade; high-grade component; receptor profile [6,13,72]	Behaviour and receptor expression are heterogeneous. Do not assume uniformly low-grade or triple-negative disease.
Mucoepidermoid carcinoma	Grade; receptor results; *MAML2* result if tested [14,15,16,17,18]	A positive *MAML2* alteration supports classification; a negative result does not exclude breast MEC. Assess risk according to histological findings.
Triple-negative invasive lobular carcinoma	Lobular morphology and cell-cohesion findings [6,11,12]	Receptor negativity does not alter lobular classification. Histological classification and predictive testing serve different purposes.

For all histological types, the report should include ER/PR results, the HER2 IHC score and ISH result when applicable, grade and stage-relevant findings where assessable, and specimen/treatment context. Histological type informs interpretation but does not independently determine treatment eligibility. Abbreviations: ER, oestrogen receptor; HER2, human epidermal growth factor receptor 2; ISH, in situ hybridisation; PR, progesterone receptor. Other abbreviations are defined in Table 1.

## Data Availability

No new experimental data were generated or analysed. The search strategies, selection counts and principal reasons for exclusion are presented in Section 2. The publications and official documents reviewed are listed in the References.
